# TIM-3 Expression Characterizes Regulatory T Cells in Tumor Tissues and Is Associated with Lung Cancer Progression

**DOI:** 10.1371/journal.pone.0030676

**Published:** 2012-02-17

**Authors:** Xin Gao, Yibei Zhu, Gang Li, Haitao Huang, Guangbo Zhang, Fengming Wang, Jing Sun, Qianting Yang, Xueguang Zhang, Binfeng Lu

**Affiliations:** 1 Department of Immunology, Institute of Medical Biotechnology, Soochow University, Suzhou, People's Republic of China; 2 Department of Immunology, University of Pittsburgh, Pittsburgh, Pennsylvania, United States of America; 3 University of Pittsburgh Cancer Institute, Pittsburgh, Pennsylvania, United States of America; 4 Jiangsu Institute of Clinical Immunology, The First Affiliated Hospital of Soochow University, Suzhou, People's Republic of China; 5 Jiangsu Province Key Laboratory of Stem Cell Research, Soochow University, Suzhou, People's Republic of China; 6 Shenzhen Clinical Center for Infectious Diseases, Shenzhen Third People's Hospital, Shenzhen, People's Republic of China; Carl-Gustav Carus Technical University-Dresden, Germany

## Abstract

**Background:**

T cell immunoglobulin-3 (TIM-3) has been established as a negative regulatory molecule and plays a critical role in immune tolerance. TIM-3 is upregulated in exhausted CD8^+^ T cells in both chronic infection and tumor. However, the nature of TIM-3^+^CD4^+^ T cells in the tumor microenvironment is unclear. This study is to characterize TIM-3 expressing lymphocytes within human lung cancer tissues and establish clinical significance of TIM-3 expression in lung cancer progression.

**Methodology:**

A total of 51 human lung cancer tissue specimens were obtained from pathologically confirmed and newly diagnosed non-small cell lung cancer (NSCLC) patients. Leukocytes from tumor tissues, distal normal lung tissues, and peripheral blood mononuclear cells (PBMC) were analyzed for TIM-3 surface expression by flow cytometry. TIM-3 expression on tumor-infiltrating lymphocytes (TILs) was correlated with clinicopathological parameters.

**Conclusions:**

TIM-3 is highly upregulated on both CD4^+^ and CD8^+^ TILs from human lung cancer tissues but negligibly expressed on T cells from patients' peripheral blood. Frequencies of IFN-γ^+^ cells were reduced in TIM-3^+^CD8^+^ TILs compared to TIM-3^−^CD8^+^ TILs. However, the level of TIM-3 expression on CD8^+^ TILs failed to associate with any clinical pathological parameter. Interestingly, we found that approximately 70% of TIM-3^+^CD4^+^ TILs expressed FOXP3 and about 60% of FOXP3^+^ TILs were TIM-3^+^. Importantly, TIM-3 expression on CD4^+^ T cells correlated with poor clinicopathological parameters of NSCLC such as nodal metastasis and advanced cancer stages. Our study reveals a new role of TIM-3 as an important immune regulator in the tumor microenvironment via its predominant expression in regulatory T cells.

## Introduction

Tumor development induces antitumor immune responses. Type 1 adaptive immune responses, mediated by Th1 cells and CTLs, are thought to be a critical component of cell-mediated immunity against cancer [1]. It has been well established, however, that many tumor infiltrating T cells are in a state of non-responsiveness due to the immune suppressive tumor microenvironment [2], [3]. Regulatory T cells and exhaustion of effector T cells are thought to be two main T cell intrinsic mechanisms that render ineffective anti-tumor immune responses [2], [4], [5]. Multiple molecular pathways, such as TGFβ, IL-10, members of B7 family, are involved in establishing the state of immune suppression within tumor [2], [6], [7], [8]. Understanding of the role of novel immune inhibitory pathways in mediating immune tolerance in the tumor microenvironment should help improving immunotherapy of cancer.

TIM-3 is expressed on Th1, Th17 cells, and CD8 T cells, but not Th2 cells [9], [10], [11]. Interaction between TIM-3 and its ligand galectin-9 inhibits Th1 and Th17 responses [12] and induces peripheral tolerance [13], [14], supporting an inhibitory role of TIM-3 in T cell responses. TIM-3 expression also identifies exhausted T cells during chronic infection. TIM-3-expressing CD4^+^ and CD8^+^ T cells produce reduced amounts of cytokine or are less proliferative in response to antigen. Blockade of the TIM-3 signaling pathway restores proliferation and enhances cytokine production in HIV-1-specific T cells [15]. Recent studies have supported an important role of TIM-3 T cell exhaustion in cancer. Tim-3 and PD-1, another marker of T cell exhaustion, are co-expressed on CD8 TILs in mice bearing transplanted tumors as well as on NY-ESO-1-specific CD8^+^ T cells in patients with advanced melanoma [4], [5]. TIM-3^+^PD-1^+^ T cells exhibit the most severe exhausted phenotype as defined by failure to proliferate and produce IL-2, TNF, and IFN-γ. Blockade of both Tim-3 and PD-1 pathways is more effective in controlling tumor growth than targeting either pathway alone, suggesting these two pathways work synergistically in establishing T cell exhaustion [4], [5].

In this study, we have investigated TIM-3 expression in TILs in none small cell lung cancer (NSCLC). We have found that TIM-3 is expressed in both CD4^+^ and CD8^+^ TILs in lung cancer tissues. Both TIM-3^+^CD4^+^ and TIM-3^+^CD8^+^ T cells produced much reduced levels of IFN-γ compared to those in TIM-3^−^CD4^+^ and TIM-3^−^CD8^+^ T cells respectively. Interestingly, TIM-3 expression on CD4^+^ T cells but not CD8^+^ T cells correlated with poor clinicopathological parameters of NSCLC such as nodal metastasis and advanced cancer stages. Strikingly, approximately 70% of TIM-3^+^CD4^+^ TILs expressed FOXP3 and about 60% of FOXP3^+^ TILs were TIM-3^+^. In contrast, TIM-3 was minimally expressed in peripheral CD4^+^ T cells, Tregs, and CD8^+^ T cells. These data suggest a novel role of TIM-3 in tumor associated regulatory T cells and its importance in human cancer progression.

## Materials and Methods

### Selection of tissue samples

A total of 51 lung cancer tissue specimens were obtained from pathologically confirmed and newly diagnosed non-small cell lung cancer (NSCLC) patients who received operation from Oct. 2009 to May 2011 in Cardiothoracic Surgery Department of First Affiliated Hospital, Soochow University in the present study. In addition, 51 cases of adjacent normal tissues from non-malignant portion were resected and chosen as controls. The normal tissues were at least 5 centimeters away from the visible tumor mass. Autologous peripheral blood mononuclear cells (PBMCs) were isolated from whole blood by Ficoll density gradient centrifugation before operation. This study was approved by the ethics committee of the First Affiliated Hospital of Soochow University. The data were analyzed anonymously and informed consent was not required.

### Harvest of tumor infiltrating lymphocytes (TILs)

Tumor tissue and adjacent normal tissue from the same patient were minced and digested with collagenase digestion solution (1 mg/ml collagenase IV (Sigma-Aldrich, St. Louis, MO, USA) in RPMI1640) at 37°C for 45 min. The pieces were then transferred to the steel mesh and single cell suspensions were obtained by mechanical grind. TILs were further purified by the gradient as per manufacturer protocol, washed and re-suspended in Hank's media for various analyses.

### Mice

6–8-week-old female C57BL/6 mice were used in tumor experiments. All animals were maintained under specific pathogen-free conditions in the animal facility at the University of Pittsburgh or Soochow University. All animal work has been approved by the Institution Animal Care and Use Committee at the University of Pittsburgh (protocol number 0906824). For tumor model experiments, mice were challenged with 2×10^5^ B16F0 cells i.d. and tumor samples were removed for analysis around day 20 as described previously [16].

### In vitro T cell stimulation

Naive T cells were purified from PBMCs of healthy donors using depleting anti-CD45RO antibody and Stemsep® human T cell enrichment kit (StemCell technologies, Vancouver, British Columbia, Canada). The purity of T cells was more than 90%. Cells were stimulated with 1 µg/ml plate-bound anti-CD3 (clone OKT3) and 2 µg/ml plate-bound anti-CD28 mAbs for up to 72 hours.

For isolating Tregs, PBMCs were stained with phycoerythrin-cyanine Dye7 (PECY7) conjugated anti-CD4 mAb (RPA-T4) and anti-CD25 phycoerythrin (PE) (BC96). A MoFlo cytometer (Beckman-Coulter, Brea, CA USA) was used to isolate CD4^+^CD25^high^ cells. These cells were then stimulated with anti-CD3 (1 µg/ml) and anti-CD28 (2 µg/ml) in the presence of IL-2 (200 U/mL, R&D Systems, Minneapolis, MN, USA). 24, 48, and 72 hours later, cells were harvested for further analysis.

### Antibodies and Flow Cytometric Analysis

Directly conjugated anti-human antibodies against the following surface molecules were used: anti-CD4 (RPA-T4) and anti-CD8 (RPA-T8) were obtained from Beckman Coulter. Anti-TIM-3 (344823), anti-PD-1(2H7), and anti-CD25 (BC96) were purchased from R&D Systems. The following monoclonal antibodies specific for mouse antigens were purchased from eBioscience (San Diego, CA, USA): CD4 (GK1.5), CD8 (53-6.7), Tim-3 (RMT3-23) and Foxp3 (FJK-16s). Flow cytometric analysis was performed using a FACS flow cytometer.

For intracellular cytokine staining, human TILs were harvested from lung cancer samples, stimulated with plate-bound anti-CD3 mAbs (1 µg/ml) and plate-bound anti-CD28 mAbs (2 µg/ml) for 16 hours and incubated for the last 3 hours with Brefeldin A (10 µg/ml, Invitrogen, Carlsbad, CA, USA). Cells were transferred to a V-bottom plate, stained with anti-CD4 or anti-CD8 in Hank's buffer (containing 1% FCS), then fixed with fixation medium, which was followed by permeabilization medium (Invitrogen, Carlsbad, CA, USA). The cells were subsequently stained with anti-IFN-γ antibody (4S.B3, Biolegend, San Diego, CA, USA). Cells producing IFN-γ were examined with flow cytometry.

For analysis of FOXP3 expression, TILs were first stained with the anti-CD4-PECY7, anti-CD25-PECY5 and anti-TIM-3-PE or anti-PD-1-PE, after fixation and permeabilization, cells were incubated with FITC-conjugated anti-human FOXP3 (259D, BioLegend, San Diego, CA, USA), based on the manufacturer's recommendations, and subjected to flow cytometric analysis.

### Statistical analysis

Statistical analyses were performed using GraphPad Prism 5.0 software package (GraphPad Software, Inc., San Diego, USA). Student's t-test, one-way ANOVA and least significant difference (LSD)-t test were used where appropriate. P-values less than 0.05 were considered as being statistically significant.

## Results

**Figure 1:**
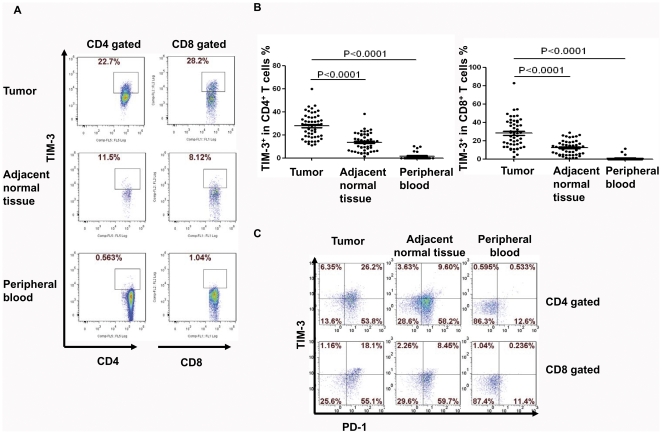
TIM-3 is highly expressed on both CD4^+^ and CD8^+^ tumor infiltrating T cells in lung cancer. Tumor infiltrating lymphocytes (TILs) were harvested from lung cancer tissues, adjacent normal tissues, and peripheral blood monouclear cells (PBMCs) from whole blood of patients. Cells were then stained for CD4, CD8, TIM-3 and PD-1. A. Representative dot-plots show TIM-3 expression on CD4^+^ and CD8^+^ T cells in various tissues from lung cancer patients. Lymphocytes were gated for further analysis of CD4 and CD8 T cells. More than 90% CD4^+^ or CD8^+^ cells were CD3^+^. B. Summarized results of the percentage (%) of TIM-3 expression on CD4^+^ and CD8^+^ T cells from lung cancer patients are shown. Horizontal bars depict the mean percentage of TIM-3 expression on CD4^+^ and CD8^+^ T cells. Error bars: s.e.m. C. Dual expression of TIM-3 and PD-1 on gated CD4^+^ and CD8^+^ T cells is shown. The p-values were calculated using the one-way ANOVA.

Since TIM-3 is involved in T cell exhaustion, we decided to investigate its expression on TILs in lung cancer specimens. Leukocytes from tumor tissues, distal normal lung tissues, and PBMC were analyzed for TIM-3 surface expression by flow cytometry. The non-specific staining was controlled by the isotype control IgG antibody (Figure S1). More than 90% of CD4^+^ cells were positive for CD3 after our purification procedure and selection of lymphocyte gate during flow cytometric analysis. In our lung cancer patient group, we have found that TIM-3 was expressed on average at about 30% of the CD4^+^ TILs and CD8^+^ TILs (Figure 1A and 1B). The percentage of TIM-3^+^CD4^+^ and TIM-3^+^CD8^+^ TILs in the distal normal lung tissues reduced to approximately 18% and 15% respectively (Figure 1A and 1B). In contrast, there was negligible expression of TIM-3 on the T cells in blood from these patients (Figure 1A and 1B). Therefore, TIM-3 expression was specifically up-regulated on T cells in the normal lung tissues and further increased in the cancer tissue. A recent report showed that, in PBMC isolated from advanced melanoma patient, a fraction of PD-1^+^ NY-ESO-1–specific CD8^+^ T cells co-expressed TIM-3 and PD-1 and these cells were more dysfunctional than TIM-3^−^PD-1^+^ and TIM-3^−^PD-1^−^ counterparts [4]. We then studied the PD-1 and TIM-3 expression on TILs from lung cancer tissues. PD-1 was expressed in more than 65% of TILs from both normal and cancer lung tissues (Figure 1C). Majority of the TIM-3^+^ TILs were PD-1^+^ in the lung tissues. In contrast, 10% of T cells in peripheral blood were positive for PD-1 (Figure 1C). Thus, we found that large numbers of TIM-3^+^PD-1^+^ T cells were enriched in lung cancer tissues. This is consistent with the fact that many of T cells in the cancer tissues are dysfunctional likely due to exhaustion or anergy.

**Figure 2:**
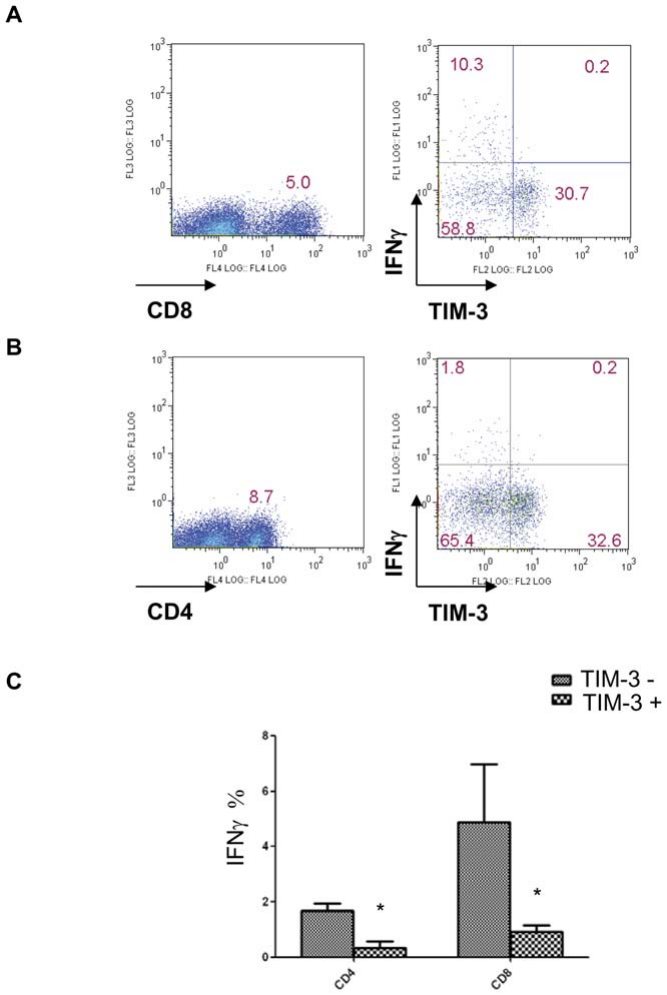
IFN-γ production by TIM-3^+^ and TIM-3^−^ T cells in lung cancer tissues. TILs were harvested from lung cancer tissue. Cells were then stimulated with plate-bound anti-CD3 mAbs (1 µg/ml) and plate-bound anti-CD28 mAbs (2 µg/ml) for 16 hours and incubated for the last 3 hours with Brefeldin A (10 µg/ml). Cells producing IFN-γ were examined with intracellular cytokine staining and flow cytometry. (A and B). Representative dot plots from one patient showed the percentage of TIM-3^+^IFN-γ^+^ and TIM-3^−^IFN-γ^+^ within CD8^+^ or CD4^+^ T cell compartment. Data shown are representative of six independent experiments. C. The average percentage of IFN-γ-producing CD8^+^ or CD4^+^ T cells among TIM-3^+^ and TIM-3^−^ fractions is shown.

Since it has been shown that antigen-specific TIM-3^+^CD8^+^ T cells are functionally exhausted in chronic infection and cancer patients, we decided to study whether TIM-3 also marked dysfunctional T cells in TILs in lung cancer tissues. TILs were stimulated with anti-CD3 and anti-CD28 for 16 h and analyzed for the IFN-γ production by flow cytometry. We have found that on average 5% TIM-3^−^CD8^+^TILs are IFN-γ^+^ upon ex-vivo stimulation with anti-CD3 (Figure 2A, 2C). In contrast, the frequency of IFN-γ producers was significantly reduced in TIM-3^+^CD8^+^ TILs (Figure 2A, 2C). Thus, our data are consistent with the idea that TIM-3 marks functional exhaustion of CD8 T cell in human cancer tissues, in keep with the recent finding using transplant mouse tumor model [5].

Besides CD8^+^ TILs, TIM-3 was found expressed on CD4^+^ TILs in human lung cancer (Figure 1) as well as in the mouse transplanted tumor [5]. Tim-3 was also highly expressed on the Th1 and Th17 cells and important for inhibiting the function of these cells [10]. Whether TIM-3 marks CD4^+^ T cell dysfunction is, however not certain. We then set out examining the frequency of IFN-γ-producing CD4^+^ TILs with respect to TIM-3 surface expression. We have found about 2% of TIM-3^−^CD4^+^ TILs were IFN-γ producers upon ex vivo stimulation with anti-CD3 (Figure 2B). In contrast, the percentage of IFN-γ^+^ cells was significantly reduced in TIM-3^+^CD4^+^ TILs (Figure 2B and 2C).

**Figure 3:**
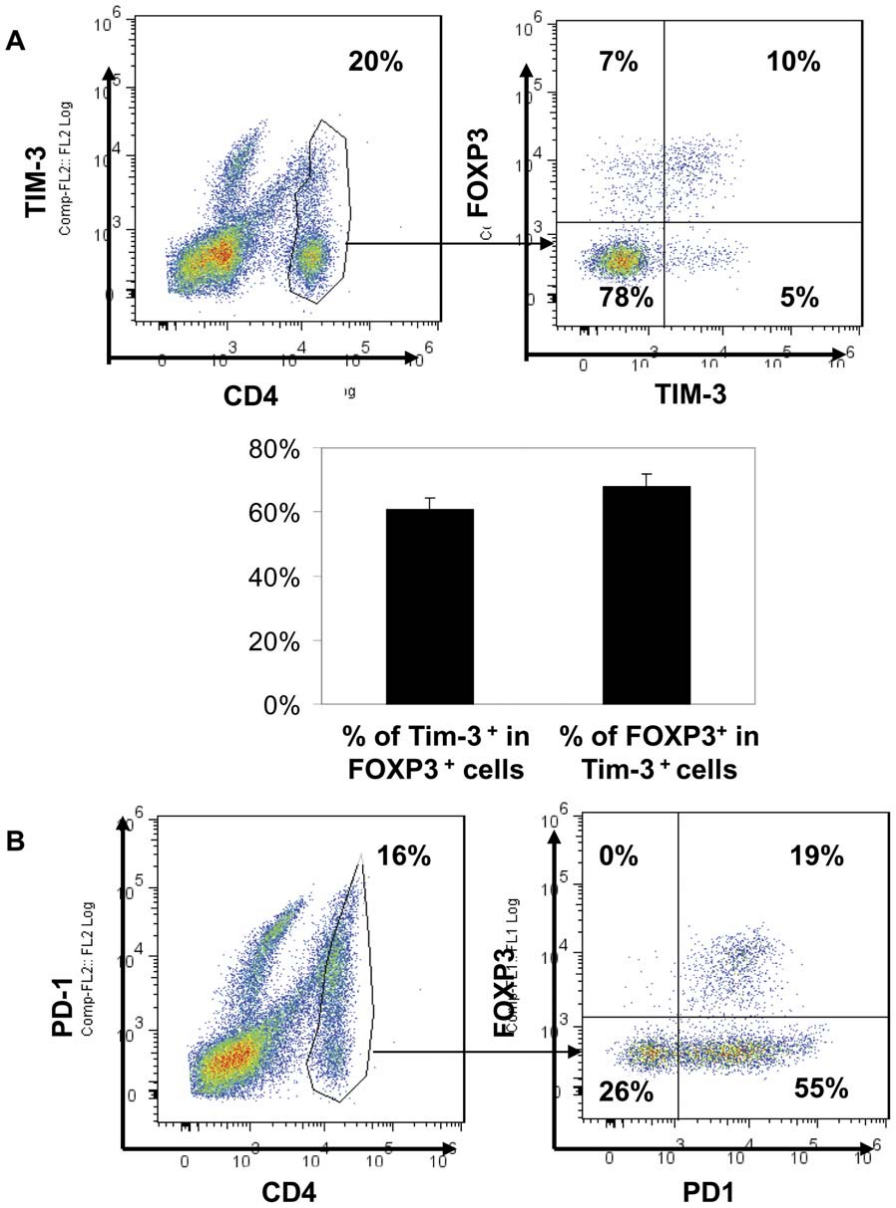
TIM-3 expression on CD4^+^ TILs and Treg. TILs were harvested from lung cancer tissue. Cells were then stained for CD4, TIM-3, and FOXP3 (A) or CD4, PD-1, and FOXP3 (B). Lymphocytes were gated for further analysis of CD4 and CD8 T cells. The percentage of each population within CD4^+^ T cell compartment was indicated. Data shown are representative of five independent experiments.

It is well established that regulatory T cells are a predominant population of CD4^+^ T cells among TILs [3]. Because Tregs are also functionally anergic, we then decided to determine whether TIM-3 is expressed in regulatory T cells among TILs using FOXP3 as the marker. To our surprise, although about 17% of the CD4^+^ TILs were FOXP3^+^ Tregs, about 70% of TIM-3^+^CD4^+^ TILs were FOXP3^+^ (Fig. 3A). In addition, about 60% of the FOXP3^+^CD4^+^ TILs expressed TIM-3 on their surfaces (Fig. 3A). Thus, TIM-3^+^CD4^+^ T cells were predominantly Tregs and most of Tregs within lung cancer TILs highly up-regulated TIM-3 expression. In line with these data, all FOXP3^+^CD4^+^ TILs express PD1 (Fig. 3B). In contrast to high expression of TIM-3 in TILs, minimal expression of TIM-3 was found on neither conventional T cells nor Tregs in peripheral blood (data not shown).

**Figure 4:**
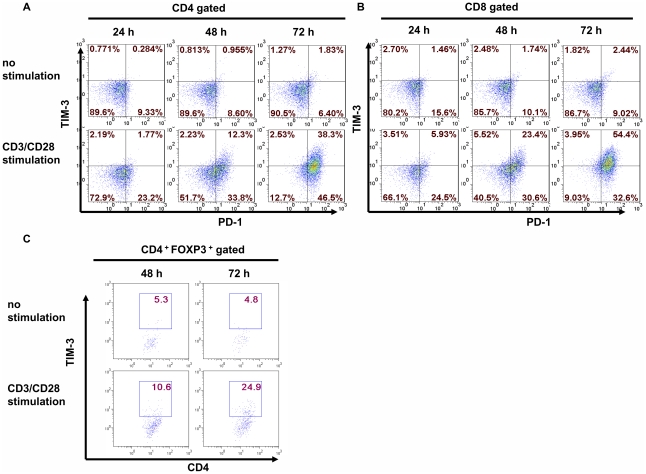
TIM-3 and PD-1 were up-regulated upon T cell activation. (A and B). Human naïve T cells were purified from PBMCs of health donors. Cells were then stimulated with plate-bound anti-CD3 plus anti-CD28 mAb. 24, 48 and 72 hours later, cells were collected and stained for CD4, CD8, TIM-3 and PD-1. The expression of PD-1 and TIM-3 was analyzed by flow cytometry on gated CD4^+^ (A) or CD8^+^ (B) T cells. C. CD4^+^CD25^high^ T cells were isolated from peripheral blood by FACS. These cells were then stimulated with anti-CD3 and anti-CD28 in the presence of IL-2 (200 U/mL). 24, 48 and 72 hours later, cells were harvested and stained for CD4, TIM-3 and FOXP3. The expression of TIM-3 on CD4^+^FOXP3^+^ T cells is shown. Data are representative of three independent experiments.

Since TIM-3 was shown to be upregulated in T cells upon in vitro culture in the presence of TCR stimulation [10], we decided to examine whether Tregs could be stimulated to express TIM-3. As a control, human CD4^+^ and CD8^+^ T cells were stimulated with anti-CD3 and anti-CD28 for 72 h. Every 24 h, we examined TIM-3 and PD-1 expression. Both PD-1 and TIM-3 were upregulated at around 48 h and further up-regulated at 72 h in both CD4^+^ and CD8^+^ T cells (Fig. 4A and 4B). We then tested whether TIM-3 could be similarly up-regulated in regulatory T cells. We enriched human Tregs by isolating CD4^+^CD25^high^ T cells using fluorescence-activated cell sorting (FACS). Then we stimulated these T cells with anti-CD3 and anti-CD28 in the presence of IL-2 for 72 h. The expression of TIM-3 and FOXP3 was examined at 48 h and 72 h. TIM-3 is expressed at negligible levels in both unstimulated naïve CD4^+^ T cells and Tregs (data not shown). Compared to unstimulated T cells, TIM-3 expression was significantly up-regulated at 48 h and further increased at 72 h (Fig. 4C). At 72 h about 25% of the FOXP3^+^ T cells were TIM-3 positive (Fig. 4C). Collectively, these data suggest that TIM-3 is up-regulated on both conventional T cells and Tregs upon stimulation via TCR.

Since TIM-3 is highly expressed in TILs isolated from lung cancer tissues, we then determined whether TIM-3 expression had any clinical significance by correlating TIM-3 expression with clinical pathological parameters. We have found the percentage of CD4^+^ and CD8^+^ T cells within TILs was not associated with any clinical pathological parameters. The ratio of CD4 versus CD8 also failed to show any clinical significance (Table 1). In addition, the frequency of TIM-3 on CD8 T cells did not show any clinical significance (Table 2). In contrast, the higher frequency of TIM-3^+^CD4^+^ TILs showed significant association with lymph node metastasis and more advanced cancer stages (Table 2). Thus, the TIM-3 expression on CD4^+^ TILs is associated with lung cancer progression.

**Table 1:** The percentage of CD4^+^, CD8^+^ T cell in TILs in patients with NSCLC.

		CD4^+^ T cell		CD8^+^ T cell		CD4^+^/CD8^+^	
	N =	mean±SD	P	mean±SD	P	mean±SD	P
**Gender**							
male	40	21.76%±10.33%		18.65%±10.86%		1.48%±0.96%	
female	11	19.33%±9.72%	0.4482	11.23%±6.38%	0.0362	1.93%±0.85%	0.168
**Age**							
≤60	19	22.12%±11.33%		18.38%±10.11%		1.52%±0.99%	
>60	32	20.63%±9.44%	0.6163	16.11%±10.77%	0.459	1.61%±0.93%	0.741
**Histological subtype**							
Squamous cell carcinoma	22	21.75%±12.20%		18.22%±11.02%		1.44%±0.88%	
Adenocarcinoma	18	20.48%±8.28%		16.36%±10.45%		1.66%±1.01%	
others	11	21.43%±9.10%	0.9263	15.57%±9.98%	0.766	1.73%±1.01%	0.657
**Tumor size**							
T1+T2	3+31	22.48%±10.64%		15.77%±8.31%		1.67%±0.90%	
T3+T4	11+6	18.80%±8.93	0.229	19.45%±13.70%	0.320	1.41%±1.02%	0.364
**Lymph node metastasis**							
N0	22	22.28%±10.09%		18.49%±10.78%		1.45%±0.73%	
N1	11	22.52%±8.41%		19.08%±10.95%		1.53%±0.83%	
N2	18	19.37%±11.31%	0.6088	14.28%±9.79%	0.353	1.74%±1.19%	0.647
**Tumor stage**							
I+II	17+10	22.40%±9.37%		18.34%±10.91%		1.51%±0.78%	
III+IV	23+1	20.05%±10.95%	0.4176	15.70%±10.04%	0.378	1.65%±1.10%	0.610

**Table 2:** Clinical significance of Tim-3^+^ TILs in patients with NSCLC.

		CD4^+^ T cells		CD8^+^ T cells	
	N =	mean±SD	P	mean±SD	P
**Gender**					
male	40	29.95%±10.17%		32.27%±15.70%	
female	11	23.85%±11.13%	0.09	24.65%±14.90%	0.156
**Age**					
≤60	19	27.83%±12.32%		27.15%±12.88%	
>60	32	29.10%±9.58%	0.682	32.70%±17.01%	0.226
**Histological subtype**					
Squamous cell carcinoma	22	27.18%±8.71%		26.51%±14.92%	
Adenocarcinoma	18	30.71%±13.07%		30.19%±11.14%	
others	11	28.14%±9.87%	0.577	39.58%±20.69%	0.075
**Tumor size**					
T1+T2	3+31	26.64%±10.29%		30.02%±16.95%	
T3+T4	11+6	32.62%±10.30%	0.056	31.84%±13.24%	0.701
**Lymph node metastasis**					
N0	22	25.06%±9.88%		28.84%±15.74%	
N1	11	27.75%±6.64%		35.37%±19.39%	
N2	18	33.53%±11.84%	0.036	29.92%±13.37%	0.525
**Tumor stage**					
I+II	17+10	25.63%±9.31%		31.06%±17.87%	
III+IV	23+1	32.00%±11.08%	0.030	30.15%±13.21%	0.839

**Figure 5:**
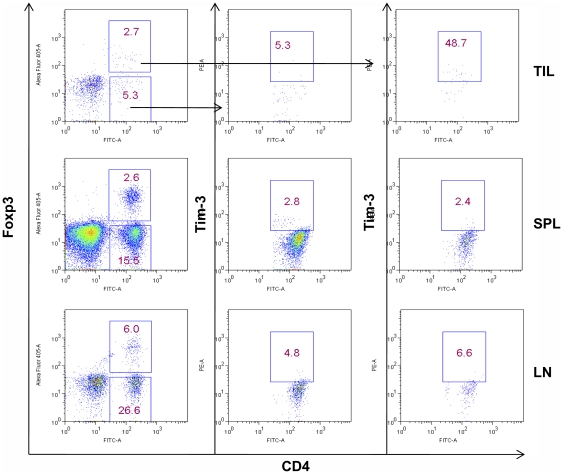
Expression of Tim-3 on TILs in mouse model of transplantable tumor. 6–8-week-old C57BL/6 mice were inoculated with 2×10^5^ B16F0 cells i.d., tumor samples, spleens, and lymph nodes were removed when tumor sizes reached around 15 mm in diameters on day 20. TILs, splenocytes, and lymph node cells were isolated for analysis by flow cytometry. Tim-3 expression on mouse CD4^+^Foxp3^+^ or CD4^+^Foxp3^−^ T cells is shown. Data shown are representative of five independent experiments.

In addition to human FOXP3^+^ TILs, we examined the Tim-3 expression on mouse Foxp3^+^ TILs. We used the B16 model because Tim-3^+^CD4^+^ TILs were recently described in this model [5]. Within the B16 tumor, we found about 50% of the Foxp3^+^CD4^+^ TILs expressed Tim-3 (Fig. 5). In contrast, Foxp3^−^CD4^+^ TILs expressed negligible levels of Tim-3 (Fig. 5). Tim-3 was expressed at minimal levels in spleen and lymph node CD4^+^ T cells irrespective of Foxp3 expression (Fig. 5). Thus, Tim-3 is predominantly present on a subset of Foxp3^+^CD4^+^ regulatory T cells in both human and mouse tumors.

## Discussion

In this study, we examined TIM-3 expression in TILs from NSCLC specimens. We found high levels of TIM-3 expression on both CD4^+^ and CD8^+^ TILs. Importantly, even though TIM-3 surface expression has been reported to indicate a functionally anergic state of CD8^+^ TILs, we found that TIM-3 expression on CD8^+^ TILs failed to associate with clinicopathological parameters in lung cancer, however the frequency of TIM-3 expression on CD4^+^ TILs did associate with lymph node metastasis and more advanced cancer stages. Further analysis revealed that TIM-3^+^CD4^+^ T cells were predominantly FOXP3^+^ and majority of FOXP3^+^CD4^+^ T cells expressed TIM-3. Our study reveals a new role of TIM-3 in the tumor microenvironment via its predominant expression in regulatory T cells.

It is interesting that we have found expression of TIM-3 on a large proportion of Tregs within lung cancer tissues. Our data mining also revealed that Tim-3 mRNA was expressed in natural Foxp3^+^CD4^+^ T cells which have been adoptively transferred to Rag1 deficient mice and Foxp3 is required for Tim-3 expression in these Treg cells (Figure S2) [17], suggesting Tim-3 expression in Tregs might be an integral part of Foxp3-regulated network within natural Tregs. We have further shown that TIM-3 is not constitutively expressed by Tregs in the human peripheral blood but can be induced upon TCR stimulation. The reason that TIM-3 is highly expressed in FOXP3^+^CD4^+^ TILs might be due to constant stimulation by tumor associated antigens within the tumor site.

The exact role of TIM-3 in the tumor infiltrating Tregs is still not known. Our data suggest that TIM-3^+^ Tregs in lung cancer tissues could be derived from natural Tregs upon chronic TCR stimulation by tumor antigens. PD-1 ligand B7-H1 and TIM-3 ligands such as galectin-9 and apoptotic cells within the tumor tissue might be important for maintaining the number and function of TIM-3^+^PD-1^+^ Tregs. In the transplantation setting, Tim-3 was shown to regulate allospecific Treg activation [14]. It was shown that Tim-3-Tim-3L-sensitive pathway was involved in the functional generation of donor-specific Tregs upon administration of tolerizing treatments [14]. Consistent with this idea, Tim-3 has recently been reported to be expressed in about 15% of Foxp3^+^Treg cells in the spleen during allo-transplantation [18]. Our study, by revealing high levels of TIM-3 expression on Tregs within human tumor, suggests that TIM-3 might play a direct role in the functional maturation of tumor infiltrating Tregs by delivering a signal within Tregs or might be important for maintaining the immunosuppressive function of these Tregs in the tumor microenvironment.

Besides natural Tregs, TIM-3 and PD-1 can also play a role in the generation of adaptive Tregs. In fact, PD-1 ligand was shown to be involved in the generation of adaptive Tregs in the tumor draining lymph nodes [19]. Galectin-9 was shown to modestly increase the Foxp3 expression in vitro model of Tregs induction by TGF-β [18], [20]. Whether the Tregs found in our studies are adaptive or natural Treg will be subject to further study.

TIM-3 has emerged as a promising target for cancer immunotherapy [21]. Recent studies have focused on the role of TIM-3 expression on CD8^+^ T cells in peripheral blood as well as within tumors [4], [5]. These study elegantly demonstrated that TIM-3 marks functionally exhausted CD8^+^ T cells and is likely responsible for failure of immunosurveillance and tumor vaccination. Our study shows that expression of TIM-3 on CD4^+^ TILs cells significantly associated with worse clinical pathological parameters in lung cancer. Therefore, TIM-3 likely plays a significant role in tumor progression by maintaining the tumor immunosuppressive environment via Tregs. Further characterization of TIM-3 expression and function within various TILs subsets should improve our knowledge of underlying mechanisms of TIM-3-mediated immunosuppression within tumor microenvironment.

## Supporting Information

Figure S1 **TIM-3 expression by flow cytometry.** Tumor infiltrating lymphocytes (TILs) were harvested from lung cancer tissues, adjacent normal tissues, and peripheral blood monouclear cells (PBMCs) from whole blood of patients. Cells were then stained for CD4 and TIM-3 or CD4 plus a control IgG antibody. Cells in the lymphocyte gate were further analyzed for CD4 and TIM-3 expression.
(PPT)

Figure S2 **Tim-3 expression in native Tregs and its dependence on Foxp3.** NCBI GEO profile database was queried for Tim-3 expression in Tregs. One result showed that Tim-3 is down-regulated in Foxp3 deficient native Tregs as shown here. DataSet Record GDS2525.
(PPT)
