# Supplementary figures and images for: TIM-3 Expression Characterizes Regulatory T Cells in Tumor Tissues and Is Associated with Lung Cancer Progression
Source: PLoS One. 2012 Feb 17;7(2):e30676. doi: 10.1371/journal.pone.0030676 (PMC3281852; doi:10.1371/journal.pone.0030676)

## Slide 1
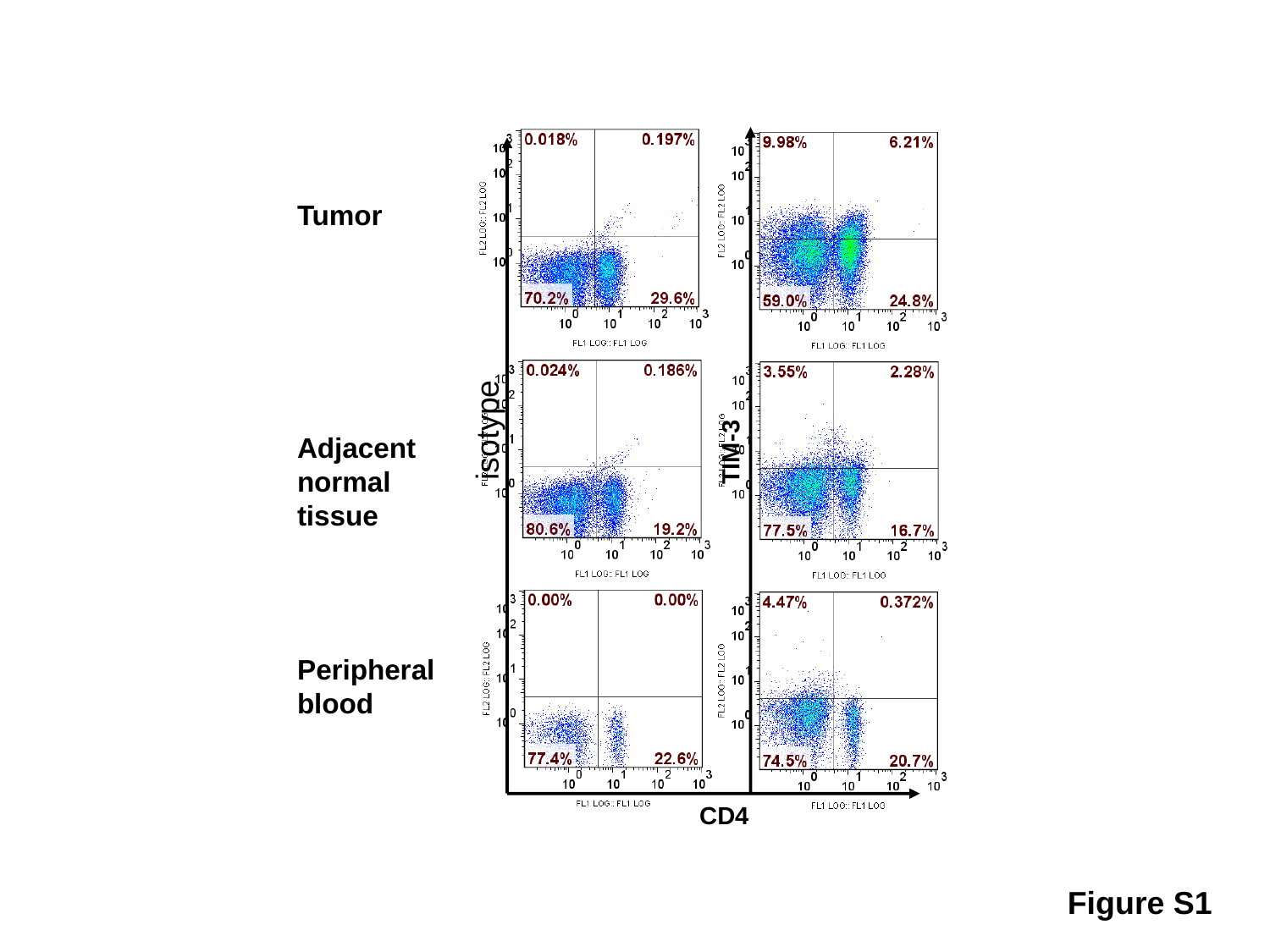

Tumor
isotype
TIM-3
Adjacent normal tissue
Peripheral blood
CD4
Figure S1

Supplement: Figure S1 — TIM-3 expression by flow cytometry. Tumor infiltrating lymphocytes (TILs) were harvested from lung cancer tissues, adjacent normal tissues, and peripheral blood monouclear cells (PBMCs) from whole blood of patients. Cells were then stained for CD4 and TIM-3 or CD4 plus a control IgG antibody. Cells in the lymphocyte gate were further analyzed for CD4 and TIM-3 expression. (PPT) [file pone.0030676.s001.ppt]

## Slide 1
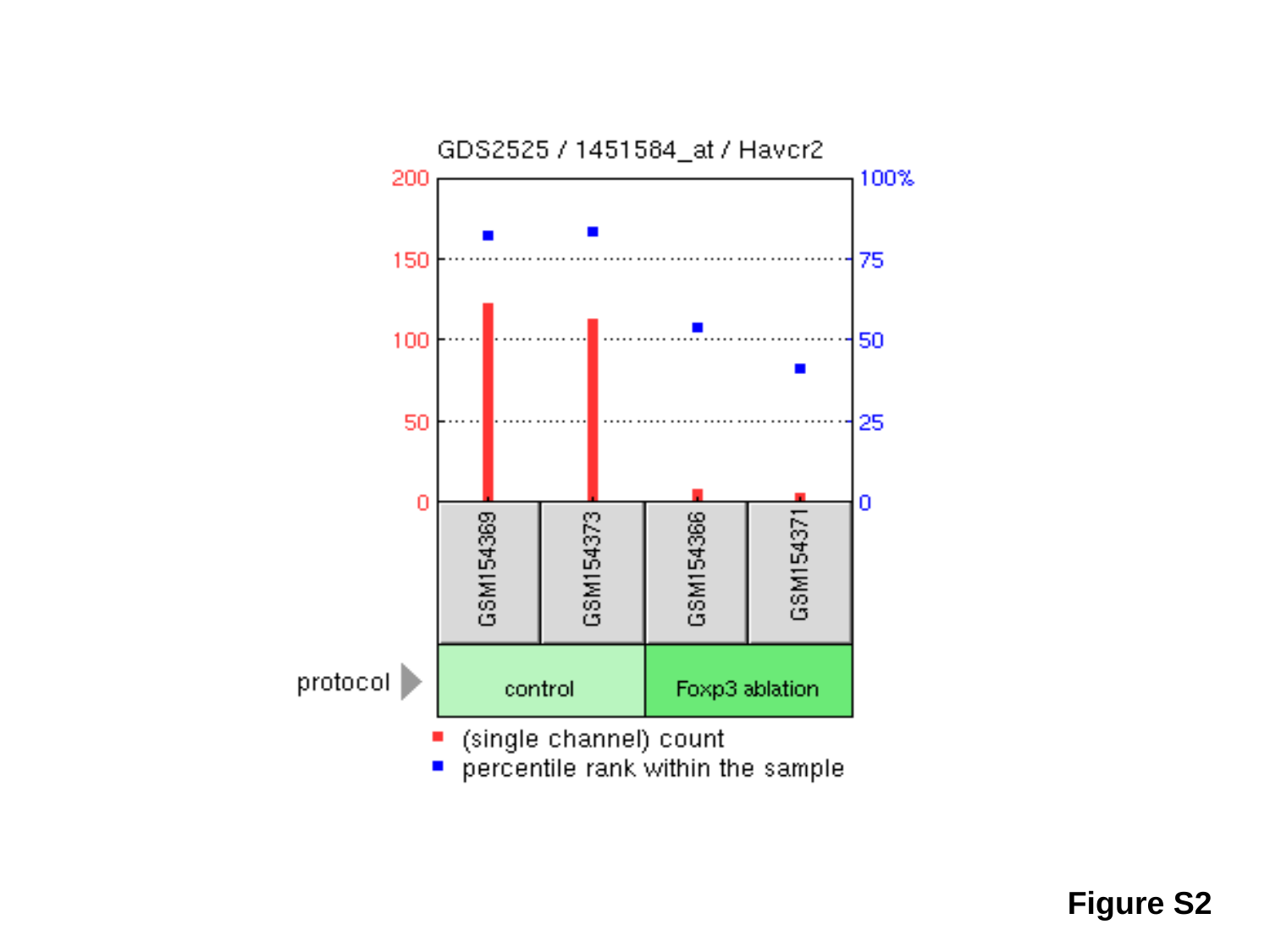

Figure S2

Supplement: Figure S2 — Tim-3 expression in native Tregs and its dependence on Foxp3. NCBI GEO profile database was queried for Tim-3 expression in Tregs. One result showed that Tim-3 is down-regulated in Foxp3 deficient native Tregs as shown here. DataSet Record GDS2525. (PPT) [file pone.0030676.s002.ppt]
